# Valsalva sinus asymmetry in bicuspid aortic valve: diameter through fused cusp is smaller than diameter through nonfused cusp but maximal diameter is the same whatever the phenotype when estimated by CMR

**DOI:** 10.1186/1532-429X-17-S1-P203

**Published:** 2015-02-03

**Authors:** Gilles Soulat, Emmanuel Messas, Arshid Azarine, Paul Achouh, Florence Pontnau, Laurence Iserin, Elie Mousseaux

**Affiliations:** Radiology, European hospital Georges Pompidou, Paris, France; Vascular medicine, European hospital Georges Pompidou, Paris, France; Paris Decartes University, Paris, France; Cardiac surgery, European hospital Georges Pompidou, Paris, France; Cardiology, European hospital Georges Pompidou, Paris, France

## Background

Previous studies establish a link between bicuspid aortic valve (BAV) morphology and aortic shape. Antero-posterior (AP) leaflets with right left (RL) fusion was found more often associated with dilatation of VS than left-right (LR) leaflets with either fusion between right and noncoronary(R-NC) cusps or between left and non-coronary cusps(L-NC). However, most of these studies were performed by transthoracic echocardiography with parasternal long axis view measurements without taking into account a possible valsalva sinus (VS) asymmetry.

The aim of our study was to assess aortic valsalva sinus size and morphology by CMR in a large cohort of BAV subjects.

## Methods

We retrospectively reviewed all aortic CMR exams of consecutive BAV subjects performed in our institution between 2006 and 2012. After exclusion of patients with prior aortic valve or root surgery and those with incomplete assessment of both aortic valve and thoracic aorta, 207 patients were included in the analysis.

Measurements of VS were performed in short axis view, perpendicular to the proximal aorta. In order to evaluate the asymmetry of VS we analysed the ratio between R-L diameter and the mean other cusp to cusp diameters; and the ratio between R-NC diameter and the mean other cusp to cusp diameters. To evaluate the size of Valsalva sinus we also proposed to estimate the maximal diameter perpendicular to the valve opening.

## Results

156 patients have AP leaflets and 51 LR leaflets. There was no difference in age between the AP and LR groups (35mm vs 35.1mm; p=0,96), in presence of a raphe ( 56% vs 43%; p=0.12), aortic valve planimetry (2.69cm2 vs 2.57 cm2 p=0.51) and regurgitant fraction in Phase Contrast imaging (6% vs 9%; p=0,75). Coarctation was present in 80 patients (42% and 27% in AP and LR group, respectively; p=0.059).

Concerning symmetry of VS; the ratio R-L/mean other sinus diameters was lower is AP leaflet group than in LR group (0.86 in AP vs 1 in LR; p<0.0001). The ratio R-NC/mean other sinus diameters was lower is LR leaflet group than in AP group (1.06 in AP vs 0.92 in LR; p<0.0001);

The maximal Valsalva sinus diameter, either side of the opening, was the same between the two group (35.6mm and 35.7mm; p=0,947), and similar results are found when analysing subgroup with or without coarctation.

LR group had higher dimension in aortic arch (23.0 vs 18.1mm; p=0.002); but similar ascending aorta ( 35.4mm for AP and 37.4mm for LR; p=0.197).

## Conclusions

Contrary to what was found in echography studies, VS diameter when estimated by CMR perpendicular to the opening, was similar between BAV opening phenotype. Futhermore the aortic diameter through the fused cusp was found smaller than the diameter through nonfused cusp.

**Figure 1:**
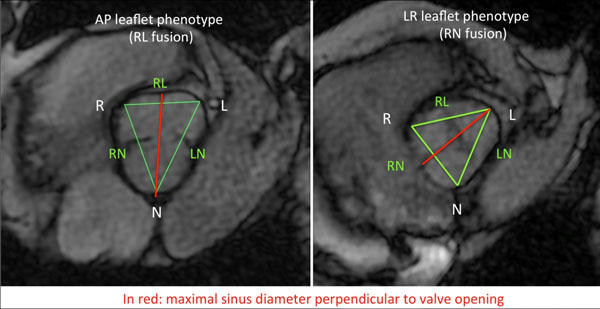
Figure 1

